# Early Weight-Bearing Following Ankle Fixation in a United Kingdom Major Trauma Centre: Real-World Adoption Versus a Contemporary Randomised Trial and British Orthopaedic Association Standards for Trauma

**DOI:** 10.7759/cureus.97076

**Published:** 2025-11-17

**Authors:** Hashim Al-Hano, Zine Beech

**Affiliations:** 1 Trauma and Orthopaedics, University Hospital Southampton NHS Foundation Trust, Southampton, GBR

**Keywords:** ankle fractures, cost-benefit analysis, early ambulation, internal fixators, length of stay, open fracture reduction, posterior malleolus, tibiofibular syndesmosis, united kingdom, weight-bearing

## Abstract

Background

Ankle fractures are common and place a substantial burden on services. Contemporary guidance generally permits weight-bearing as tolerated after stable fixation with early outpatient review. Recent randomised evidence suggests that beginning weight-bearing at around two weeks after open reduction and internal fixation (ORIF) can achieve at least comparable functional outcomes without increased complications and may be resource-efficient. Despite this, the adoption of early weight-bearing remains variable. We evaluated our centre’s timing to first weight-bearing and early safety in the year following the dissemination of new evidence.

Methods

We conducted a single-centre retrospective cohort study at a UK major trauma centre (September 2024-August 2025). Adults (≥18 years) undergoing ankle ORIF were included; exclusions were hindfoot nails, tibial plafond (pilon) fractures, open fractures, or missing follow-up. Data sources were the local trauma database, operative notes, discharge summaries, fracture-clinic letters, imaging, and general practitioner records. Variables included age, sex, length of inpatient stay (LOS), fracture pattern (unimalleolar/bimalleolar/trimalleolar), posterior malleolus fixation (yes/no), syndesmosis fixation (none/screw/suture-button), and discharge device (cast/boot). The primary outcome was time to first weight-bearing (bands: at 2 weeks, 2-6 weeks, >6 weeks; sub-bands 6-8 and >8 weeks). Safety within eight weeks comprised unplanned emergency department/clinic contact, re-operation, and radiographic loss of reduction. Analyses used descriptive statistics (mean/standard deviation (SD); median/interquartile range (IQR)); between-group comparisons employed Kruskal-Wallis or Mann-Whitney U for days to weight-bearing and chi-square for proportions >6 weeks (two-sided p<0.05). Continuous outcomes (LOS, days to first weight-bearing) were non-normally distributed (Shapiro-Wilk p<0.001); hence, non-parametric tests were used.

Results

Forty-two patients were included. The mean age was 51.1 years (SD 16.5), with a median age of 49.5 years (IQR 39.2-62.5). LOS had a mean of 7.0 days (SD 7.9) and a median of 3.5 days (IQR 1.0-13.0). Time to first weight-bearing: at 2 weeks 2/42 (4.8%), 2-6 weeks 9/42 (21.4%), >6 weeks 31/42 (73.8%) (including 6-8 weeks 21/42 (50.0%), >8 weeks 10/42 (23.8%)). Safety ≤8 weeks in the 6-week or longer group showed unplanned contact 1/42 (2.4%), re-operation 0/42, loss of reduction 0/42, and delayed union 2/42 (4.8%). Safety margins in the 2-week group did not show any complications (0/42 in all parameters). Days to first weight-bearing did not differ significantly by fracture pattern (p=0.066) or syndesmosis fixation (p=0.383); posterior malleolus fixation was associated with longer time (Mann-Whitney p=0.036). Proportions exceeding six weeks did not differ significantly across subgroups.

Conclusions

Early weight-bearing after ankle ORIF was seldom implemented locally, with most patients first weight-bearing at ≥6 weeks despite reassuring short-term safety. In light of the recent clinical guidance, a default “weight-bearing as tolerated from two weeks” pathway (with clearly defined exceptions), standardised discharge/clinic instructions, and planned re-audit may improve implementation without compromising safety.

## Introduction

Ankle fractures are common injuries that place a considerable burden on patients and services [1,2]. The British Orthopaedic Association Standards for Trauma (BOAST) recommend that most patients should be allowed to bear weight as tolerated after stable fixation, with follow-up arranged within six weeks [3]. More recently, the United Kingdom (UK) Weight-bearing in Ankle Fractures (WAX) randomised controlled trial compared commencing weight-bearing at two weeks versus six weeks after ankle open reduction and internal fixation (ORIF), reporting non-inferior, if not slightly superior, functional outcomes with early weight-bearing, no increase in complications, and a high probability of cost-effectiveness for the National Health Service [4,5]. Despite this guidance and trial evidence, adoption of early weight-bearing appears variable across centres [6].

Against this backdrop, we evaluated our centre’s real-world timing to first weight-bearing and early safety outcomes in relation to BOAST recommendations and the WAX trial. We deliberately selected a study window from September 2024 to August 2025 to capture practice patterns after the WAX publication (late June 2024), allowing a reasonable period for local dissemination and potential implementation.

## Materials and methods

Design and setting

Retrospective observational cohort study conducted at a UK major trauma centre from September 2024 to August 2025.

Participants

Adults aged ≥18 years undergoing ankle ORIF were included. Exclusions were hindfoot nails, tibial plafond (pilon) fractures, open fractures, or missing follow-up. These criteria excluded 21 patients from the cohort.

Data sources

Patients were identified using the local trauma database (eTrauma). Their eligibility for this review and their management were then verified using CHARTS, the Trust's electronic record and confirmed using SECTRA PACS (Sectra AB, Linköping, Sweden), operative notes, discharge summaries, and fracture-clinic letters.

Variables

We collected data on age, sex, and length of inpatient stay (LOS); fracture pattern (unimalleolar, bimalleolar, or trimalleolar); posterior malleolus fixation (yes or no); syndesmosis fixation (none, screw, or suture-button using the TightRope™ XP implant system by Arthrex); and discharge device (cast or boot).

Outcomes

Time to first weight-bearing was categorised into primary bands (at 2 weeks, 2-6 weeks, and >6 weeks) and sub-bands (6-8 weeks and >8 weeks). Weight-bearing status was observed during follow-up clinic visits. Safety parameters assessed within eight weeks included unplanned emergency department or clinic contact, re-operation, and radiographic loss of reduction.

Pre-specified notes

Seven patients underwent staged external fixation prior to ORIF; two had Maisonneuve injuries; two were instructed to weight-bearing as tolerated (WBAT) at two weeks, but were documented at 15-16 days due to clinic scheduling and were retained in the "at 2-week" band by intention.

Statistical analysis

Descriptive statistics are reported as mean with standard deviation (SD) and median with interquartile range (IQR). Continuous outcomes (LOS, days to first weight-bearing) were non-normally distributed (Shapiro-Wilk p<0.001); hence, non-parametric tests were used. Between-group comparisons for days to first weight-bearing/WBAT used Kruskal-Wallis (three or more groups) or Mann-Whitney U (two groups). Comparisons of the proportion with >6 weeks to first weight-bearing used chi-square tests (exploratory, given small sample/event counts). Two-sided p < 0.05 was considered statistically significant. Analyses were performed on complete cases; no imputation was undertaken.

Ethics and reporting

The project was conducted as a service evaluation/retrospective cohort using routinely collected data and did not involve any change to clinical care. Institutional guidance indicated that formal research ethics committee review was not required; patient consent was not required for anonymised retrospective analysis. Reporting followed the Strengthening the Reporting of Observational Studies in Epidemiology (STROBE) recommendations where applicable [7].

## Results

Cohort (September 2024-August 2025)

A total of 42 patients were included. The mean age was 51.1 years (± SD 16.5), with a median of 49.5 years (IQR 39.2-62.5). The mean LOS was 7.0 days (± SD 7.9), with a median of 3.5 days (IQR 1.0-13.0). There were 16 male patients (38.1%) and 26 female patients (61.9%). Fracture patterns comprised 11 unimalleolar, 19 bimalleolar, and 12 trimalleolar injuries. Syndesmosis fixation included no fixation in 13 patients, screw fixation in 19, and suture-button fixation (TightRope™) in 10. Posterior malleolus fixation was performed in eight patients and not performed in 34. At discharge, 41 patients were placed in a cast and one in a boot, with nearly all patients discharged postoperatively in a back slab (n = 41) (Table 1).

**Table 1:** Cohort characteristics (September 2024 to August 2025) Values are presented as mean with standard deviation or median with interquartile range (IQR), as appropriate. Proportions are shown as a number over the total with a percentage. Length of inpatient stay refers to days from operation to discharge.“Suture-button” refers to a TightRope device by Arthrex. “Back slab” indicates a posterior splint applied at discharge.

Variable	Value
Cohort size	42 patients
Age, mean ± SD, years	51.1 ± 16.5
Age, median (IQR), years	49.5 (39.2-62.5)
Length of inpatient stay (LOS), mean ± SD, days	7.0 ± 7.9
LOS, median (IQR), days	3.5 (1.0-13.0)
Sex, male, n/N (%)	16/42 (38.1)
Sex, female, n/N (%)	26/42 (61.9)
Fracture pattern - unimalleolar	11
Fracture pattern - bimalleolar	19
Fracture pattern - trimalleolar	12
Syndesmosis fixation - none	13
Syndesmosis fixation - screw	19
Syndesmosis fixation - suture-button (TightRope™)	10
Posterior malleolus fixation - yes	8
Posterior malleolus fixation - no	34
Discharge device - cast	41
Discharge device - boot	1
Back slab at discharge (post-operative)	41/42 patients

Time to first weight-bearing

At 2 weeks in 2 of 42 patients (4.8%) (both of whom had intended 2-week WBAT or full weight-bearing documented in the operation note but converted at 15-16 days due to fracture-clinic scheduling) at 2-6 weeks in 9 of 42 patients (21.4%), and at more than 6 weeks in 31 of 42 patients (73.8%). Within the >6-week group, 21 of 42 patients (50.0%) began weight-bearing at 6-8 weeks and 10 of 42 (23.8%) at >8 weeks. For completeness, the cohort’s mean time to first weight-bearing was 52.1 days, with a median of 48 days (IQR 42-56) (Tables 2-3).

**Table 2:** Primary weight-bearing bands after ankle open reduction and internal fixation Bands are defined as weight-bearing documented at two weeks, between two and six weeks, and after six weeks post-operation. The “at two weeks” category includes two intention cases recorded at 15-16 days due to clinic scheduling. Percentages are calculated from the total cohort (N=42) and may round to 100%. Terminology is aligned with the British Orthopaedic Association Standards for Trauma (BOAST) guidance and used consistently across the manuscript.

Weight-bearing band (primary)	Count	Percent
At 2 weeks	2	4.8
2-6 weeks	9	21.4
>6 weeks	31	73.8

**Table 3:** Sub-bands for time to first weight-bearing after ankle open reduction and internal fixation Sub-bands are defined as weight-bearing documented at two weeks, two to four weeks, four to six weeks, six to eight weeks, and after eight weeks post-operation. The “at two weeks” category includes two intention cases recorded at 15-16 days due to clinic scheduling. Percentages are calculated from the total cohort (N=42) and may round to 100%.

Weight-bearing band (sub-band)	Count	Percent
At 2 weeks	2	4.8
2-4 weeks	0	0
4-6 weeks	9	21.4
6-8 weeks	21	50
>8 weeks	10	23.8

Safety within eight weeks

In the >6-week group, unplanned emergency department or clinic contact occurred in 1 of 42 patients (2.4%); there were no re-operations (0/42, 0%), while delayed union was observed in 2 of 42 patients (4.8%). In the 2-week group, no safety concerns were found (Table 4).

**Table 4:** Early safety outcomes within eight weeks of ankle open reduction and internal fixation Outcomes were assessed at eight weeks post-operation using clinic letters, discharge summaries, and imaging reports. “Unplanned contact” includes unscheduled emergency department or fracture-clinic attendance. “Re-operation” denotes any return to theatre. “Radiographic loss of reduction” reflects displacement on follow-up imaging judged by the treating team. “Delayed union” indicates delayed clinical or radiographic progression documented by the surgeon within the eight-week window. Proportions are shown as a number over the total with a percentage (N=42).

Result	Value
Safety ≤8 weeks: unplanned contact n/N (%)	1/42 (2.4)
Safety ≤8 weeks: re-operation n/N (%)	0/42 (0.0)
Safety ≤8 weeks: radiographic loss of reduction n/N (%)	0/42 (0.0)
Safety ≤8 weeks: delayed union n/N (%)	2/42 (4.8)

Between-group comparisons (exploratory)

For days to first weight-bearing, there were no statistically significant differences by fracture pattern (Kruskal-Wallis p = 0.066) or by syndesmosis fixation (p = 0.383). Posterior malleolus fixation was associated with a longer time to first weight-bearing (Mann-Whitney U p = 0.036). For the proportion of patients taking more than 6 weeks to achieve first weight-bearing, chi-square tests showed no statistically significant differences across subgroups: fracture pattern p = 0.051, syndesmosis fixation p = 0.760, posterior malleolus fixation p = 0.154, and sex p = 1.000 (Table 5).

**Table 5:** Statistics summary Non-parametric tests were used for skewed data. Kruskal-Wallis reports H for “days to weight-bearing”; Mann-Whitney reports U for posterior malleolus fixation (yes vs. no); chi-square reports χ^2^ for the proportion with >6 weeks to first weight-bearing. Two-sided p < 0.05 was considered significant; analyses were exploratory (no multiple-comparison adjustment). Degrees of freedom for χ^2^: fracture pattern df=2, syndesmosis fixation df=2, posterior malleolus fixation df=1, sex df=1.

Comparison	Test	Statistic (H/U/χ^2^)	P-value
Days to weight-bearing by fracture pattern	Kruskal-Wallis	5.431	0.066
Days to weight-bearing by syndesmosis fixation	Kruskal-Wallis	1.921	0.383
Days to weight-bearing by posterior malleolus fixation (Y vs. N)	Mann-Whitney U	202	0.036
% >6 weeks by fracture pattern	Chi-square	5.962	0.051
% >6 weeks by syndesmosis fixation	Chi-square	0.549	0.760
% >6 weeks by posterior malleolus fixation	Chi-square	2.033	0.154
% >6 weeks by sex	Chi-square	0.000	1.000

## Discussion

This real-world cohort demonstrates very delayed progression to weight-bearing compared with contemporary guidance and trial evidence. BOAST supports WBAT after stable fixation, and the WAX trial showed that weight-bearing from two weeks is safe, functionally non-inferior (slightly superior), and likely cost-saving [3-5]. In our centre, 73.8% first weight-bearing after six weeks, only 4.8% are weight-bearing at two weeks, and none are discharged WBAT. Early safety here was reassuring (2.4% unplanned contact; no re-operations; no radiographic losses, and 4.8% delayed union in the delayed weight-bearing group and none to report for the 2-week early weight-bearing group), consistent with the WAX findings and recent meta-analyses supporting earlier mobilisation [8,9].

Decision-making around the posterior malleolus and the syndesmosis in our unit reflects multidisciplinary team (MDT) discussion, intra-operative stability assessment, patient factors (age, bone quality, soft-tissue status, comorbidity), and surgeon experience [10]. In this context, it is unsurprising that posterior malleolus fixation was associated with longer time to first weight-bearing in our dataset: It often co-occurs with more complex fracture patterns, staged external fixation, and a more cautious post-operative pathway [11,12]. We interpret the statistically significant difference in days to weight-bearing for posterior malleolus fixation as a marker of case-mix and pathway caution rather than a causal effect of the fixation itself. Notably, when put as ">6 weeks" versus "≤6 weeks", between-group differences were not significant - again suggesting a shift in central tendency rather than a categorical change in practice.

Beyond clinical safety, WAX highlights pragmatic benefits of an early-weight-bearing pathway: faster return to function, earlier recovery of independence, and high probability of cost-effectiveness driven by reduced resource use (e.g., fewer clinic visits and imaging triggered by cast-related issues, less physiotherapy intensity) and earlier return to work for many patients [5]. Our findings include very low early complication rates despite predominantly delayed weight-bearing, which supports the feasibility of moving earlier without compromising safety, provided fixation is stable and soft tissues are satisfactory.

Implementation barriers in our setting likely include entrenched surgeon preference, default casting at discharge, concern regarding syndesmosis screws [13,14], and operational factors such as clinic scheduling that delay cast change or boot fitting. A pragmatic approach would be to adopt a default “WBAT from two weeks” when intra-operative stability criteria are met, with clear exceptions (unstable fixation, neuropathy/insensate foot, poor soft-tissue envelope, patient non-adherence risk). To operationalise this, we propose (1) presenting these data at our local trauma MDT/audit meeting to agree indications and exceptions; (2) issuing a one-page protocol and checklist for theatre, recovery, and discharge (including standardised operation-note phrases and discharge instructions); (3) default booking of a two-week clinic slot for cast change/boot and documented weight-bearing status; (4) templated clinic letters that state WBAT unless an exception is ticked; (5) staff and patient education materials; and (6) ensuring boot availability on the ward and/or theatres.

Additionally, we recommend a plan-do-study-act (PDSA) cycle with re-audit at six to nine months, using the same metrics (time to first weight-bearing bands, unplanned contact, re-operation, radiographic loss of reduction) and simple balancing measures (falls, cast/boot issues). This will test whether an agreed protocol closes the implementation gap and maintains the excellent early safety profile observed here.

Finally, BOAST has recently updated its generic guidance to use clearer terms for post-operative loading (unrestricted, limited, and non-weight-bearing) [15]. To avoid confusion and maintain consistency with ankle fracture-specific guidance and the evidence we reference (including WAX), we have retained the ankle fracture BOAST terminology in this study (which includes weight bearing as tolerated).

Limitations

This study has several limitations. First, it is a retrospective, single-centre cohort with a modest sample size (N=42), limiting precision, statistical power, and generalisability. Second, weight-bearing status and dates were abstracted from operation notes, discharge summaries, and clinic letters; they reflect documented instructions rather than continuously observed behaviour, so misclassification is possible. Relatedly, clinic scheduling (e.g., first review at 15-16 days) may have systematically delayed documentation of “at two weeks”, introducing timing bias.

Third, treatment decisions - including posterior malleolus and syndesmosis fixation - were influenced by multidisciplinary discussion, intra-operative stability, patient factors, and surgeon preference, so confounding by indication is likely. Given sample size constraints, we used primarily univariable, non-parametric comparisons without multivariable adjustment, and we did not correct for multiple testing; subgroup findings should therefore be considered exploratory.

Fourth, radiographic outcomes were limited to early follow-up; we did not assess medium- or long-term function, malreduction metrics, implant failure, or patient-reported outcomes. Finally, we did not perform a formal economic analysis; comments on potential cost savings draw on external evidence rather than cost data from our centre.

## Conclusions

In this single-centre cohort, early weight-bearing after ankle ORIF is rarely implemented: most patients are instructed to first weight-bear at or beyond six weeks despite reassuring short-term safety. Our findings contrast with contemporary guidance and trial evidence (BOAST and WAX) that support weight-bearing from two weeks without increased complications and with probable cost savings. The longer time to weight-bearing observed in patients with posterior malleolus fixation likely reflects case-mix and pathway caution rather than a causal effect.

Adopting a default “WBAT from two weeks” pathway - when fixation is stable and soft tissues permit - together with clear exceptions, standardised discharge/clinic instructions, and early clinic scheduling, offers a practical route to align practice with the evidence. We plan to present these data at the trauma multidisciplinary meeting to support implementation and to re-audit using the same metrics after six to nine months. While limited by retrospective design, modest sample size, and reliance on documented instructions, these results suggest that earlier mobilisation is achievable locally without compromising safety and may reduce system costs.
